# Pathophysiology of heart failure and frailty: a common inflammatory origin?

**DOI:** 10.1111/acel.12581

**Published:** 2017-03-07

**Authors:** Lavanya Bellumkonda, Daniel Tyrrell, Scott L. Hummel, Daniel R. Goldstein

**Affiliations:** ^1^ Section of Cardiovascular MedicineDepartment of MedicineYale School of MedicineNew HavenCTUSA; ^2^ Section of Cardiovascular MedicineDepartment of MedicineUniversity of MichiganAnn ArborMIUSA; ^3^ Ann Arbor Veterans Affairs Healthcare SystemAnn ArborMIUSA; ^4^ Institute of GerontologyUniversity of MichiganAnn ArborMIUSA

**Keywords:** frailty, heart failure, pathophysiology

## Abstract

Frailty, a clinical syndrome that typically occurs in older adults, implies a reduced ability to tolerate biological stressors. Frailty accompanies many age‐related diseases but can also occur without overt evidence of end‐organ disease. The condition is associated with circulating inflammatory cytokines and sarcopenia, features that are shared with heart failure (HF). However, the biological underpinnings of frailty remain unclear and the interaction with HF is complex. Here, we describe the inflammatory pathophysiology that is associated with frailty and speculate that the inflammation that occurs with frailty shares common origins with HF. We discuss the limitations in investigating the pathophysiology of frailty due to few relevant experimental models. Leveraging current therapies for advanced HF and current known therapies to address frailty in humans may enable translational studies to better understand the inflammatory interactions between frailty and HF.

## Introduction

As the number of people over 65 year of age grows, so too will the rate of cardiovascular diseases in the population. This growth will pose an increasing burden on our healthcare system and resources, particularly as cardiovascular disease in older people overshadows all other age‐related end‐organ diseases including infectious diseases, cancer, and lung diseases. There is thus an urgent need to understand how aging impacts cardiovascular diseases. Among cardiovascular diseases in older people, heart failure (HF) is a major cause of morbidity and mortality. Importantly, people over 65 years of age constitute >80% of patients with HF and the incidence of HF is 10 per 1000 in people aged >65 years of age (Go *et al*., 2013).

Approximately 25% of older patients with HF exhibit evidence of frailty, a clinical syndrome characterized by reduced tolerance to biological stressors (Boxer *et al*., 2008; Dodson & Chaudhry, 2012). Frailty can also occur independently of HF, although both frailty and HF are associated with a proinflammatory phenotype. It was recently demonstrated that of several organ systems, frailty is most highly associated with cardiovascular dysfunction (Nadruz *et al*., 2016). In this review, we speculate that HF and frailty may be linked via a common inflammatory pathway and describe how this inflammation may be initiated. We discuss our limitations in understanding the underlying pathophysiology of frailty posed by current confines in experimental models of frailty in contrast to HF (Patten & Hall‐Porter, 2009).

## Definition of frailty

Frailty is a state of excess vulnerability to biological stressors due to a decline in functional reserve across multiple physiologic systems with a resultant inability to maintain homeostasis at baseline or regain homeostasis after a destabilizing event (Xue, 2011). While there is no universally accepted method of assessing frailty, some of the commonly used tools include individual metrics of unintentional weight loss, fatigue, low physical function, and strength such as gait speed and handgrip strength (Ling *et al*., 2010), and presence of comorbidities. Comprehensive scales such as the Fried index, Rockwood index, and Short Physical Performance Battery (SPPB) are also commonly used (Afilalo *et al*., 2009, 2014; Dodson & Chaudhry, 2012; Flint *et al*., 2012; Morley *et al*., 2013), described in detail elsewhere (Fried *et al*. 2001; Morley *et al*., 2013; Chamberlain *et al*., 2016). Together, these serve to limit mobility, independence, and the ability to perform activities of daily living in older adults (Chamberlain *et al*., 2016). In this review, we describe the underlying inflammatory phenotype associated with frailty and speculate how it may be linked to the inflammatory phenotype that occurs with HF.

Whether frailty is an independent process or the result of a culmination of various comorbidities that occur with aging is an ongoing debate. Epidemiological data from the Cardiovascular Health Study suggests that 15% of prefrail and 7% of frail patients do not exhibit comorbid conditions (Fried *et al*. 2001). This finding supports the concept that frailty and other diseases are independent conditions. It is also possible that there may be two forms of frailty, one associated with aging and resultant sarcopenia and anorexia but without evidence of end‐organ disease, and the other more common form, which occurs in the setting of comorbidities or severe illnesses (Fried *et al*. 2001).

End‐organ disease such as HF exacerbates frailty. It is unclear, however, if frailty itself can contribute to organ dysfunction and resultant HF. Results from the Health, Aging, and Body Composition Study found that in community‐dwelling individuals, moderate and severe frailty have an increased risk of incident heart failure diagnosis (Khan *et al*., 2013). While there is no direct relationship between frailty and severity of HF, there is evidence that frailty is more common with acute decompensated heart failure (ADHF) as compared to those with chronic stable preserved ejection fraction (HFpEF) and reduced ejection fraction (HFrEF; Reeves *et al*., 2016). More than half of the patients admitted with ADHF were found to be frail. This state is promoted by chronic underlying skeletal muscle changes from long‐standing heart failure, but an acute myopathic process can also develop during hospitalization and be compounded by immobility (Puthucheary *et al*., 2013; Kitzman *et al*., 2014). Our lack of understanding of the etiology and the implications of frailty are due to our poor comprehension of the pathophysiology of this condition. Frailty and HF are both associated with inflammation (Fedarko, 2011), thus gaining a better understanding of how inflammation occurs may reveal how frailty develops and how it relates to HF.

## Overview of the pathophysiology of inflammation

### Innate immune signaling

The induction of inflammation is a host defense mechanism evolved to alert the immune system and limit microbial invasion. Inflammation is typically induced when the innate immune system, which acts as the first line of defense to infection (Medzhitov & Janeway, 2000), is activated. The Toll‐like receptors (TLR) are membrane‐bound innate immune receptors that respond to microbial components, for example, cell surface‐bound TLR4 is activated by lipopolysaccharide, and TLR9 expressed within endosomes is activated by unmethylated CpG DNA sequences (reviewed in Takeuchi & Akira, 2010). Activation of TLRs leads to the translocation of NF‐kB, a key intracellular controller of inflammation, leading to the production of inflammatory cytokines (e.g., IL‐6 and TNF‐α) and the upregulation of costimulatory molecules on dendritic cells and macrophages, and key innate sentinel immune cells (Shirali & Goldstein, 2008). In addition to membrane‐bound innate immune receptors, intracellular innate immune pathways include the inflammasome, a multiprotein complex that is activated by Nod‐like receptors (NLR) which leads to the production of IL‐1β (Chen & Nunez, 2010). The inflammasome is known to be activated by bacteria (e.g., salmonella) and viruses (e.g., influenza; Lamkanfi & Dixit, 2014). The RIG‐I‐like receptors (RLR) respond to intracellular nucleic acids in the context of viral infections (e.g., influenza virus) to produce type 1 interferons (IFN; Yoneyama *et al*., 2015).

### Sterile inflammation

The TLRs and inflammasome pathways are activated by host‐derived proteins in addition to microbial motifs leading to sterile inflammation, which is defined as inflammation without microbes (Shen *et al*., 2013). Sterile inflammation occurs in conditions such as acute ischemia reperfusion injury or during chronic inflammatory processes evident with HF (Shen *et al*., 2013). Chronic inflammatory markers are also associated with frailty (Darvin *et al*., 2014). Sterile inflammation occurs when cellular necrosis leads to the release of host substances that typically do not activate the innate immune system. For example, the release of mitochondrial DNA that activates Toll‐like receptor (TLR) 9 induces myocardial inflammation and HF (Oka *et al*., 2012). Free mitochondrial DNA in plasma can also activate TLR5 and the NLR‐induced inflammasome (Zhang *et al*., 2010; Dall'Olio *et al*., 2013). Sterile and microbial inflammation can act simultaneously as exemplified by HF when reduced gut perfusion compromises epithelial surfaces and leads to gut commensal bacteria translocating into the portal circulation. Once in the portal circulation, these bacteria active innate immune receptors, such as TLRs, to induce inflammation.

Whether the initial stimulus is microbial, sterile, or a combination of both, persistent innate immune activation leads to chronic inflammation, which can manifest as increased levels of circulating levels of pro‐inflammatory cytokines such as TNF‐α, IL‐6, and IFN‐γ, and proteins such as C‐reactive protein (CRP). Importantly, the induction of inflammation leads to negative feedback pathways to resolve inflammation, via immune‐suppressive cytokines like IL‐10 or resolvins (Serhan *et al*., 2008). These mediators enhance the clearance of dying neutrophils by macrophages, a process termed efferocytosis (Greenlee‐Wacker, 2016). Hence, chronic inflammation can occur either by persistent activation or a failure to resolve inflammation.

Aging in humans has been associated with increased, low levels of circulating pro‐inflammatory cytokines (Tracy, 2013; De Martinis *et al*., 2006).Even in reportedly healthy older humans, there is evidence for an increase in circulating inflammatory proteins. The precipitants of chronic, low‐grade inflammation with aging could be sterile or microbial. Sterile inflammation may result from the breakdown of tissues such as adipose, skeletal muscle, or cardiomyocytes, whereas chronic, indolent viral infections (e.g., cytomegalovirus) can also lead to chronic inflammation. This may be exacerbated in HF as increased intravascular pressure may result in intestinal congestion, abdominal discomfort, and appetite loss which may lead to cachexia (Valentova *et al*., 2016). Frailty is associated with increased circulating of TNF‐α, IL‐6, IFN‐γ, and CRP, and these mediators are also elevated in HF patients (Kalogeropoulos *et al*., 2010; Mann, 2015). This suggests that there could be shared inflammatory pathways that are activated by HF and frailty, as discussed below. In support of the inflammatory pathophysiology of frailty, a recent study found that nonagenarians, but not humans under 30 years of age, exhibit a significant correlation between plasma levels of cell‐free DNA, a known activator of TLR9 and elevated levels of IL‐6 and CRP (Jylhava *et al*., 2013). Importantly, this study correlated levels of plasma DNA with frailty in the older patient cohort.

## How aging, frailty, and HF interact to induce inflammation

How aging occurs remains unclear (Gladyshev, 2016). Current theories include: genetic programing, mutation accumulation, antagonistic pleiotropy (a process in which genes that are beneficial for reproductive fitness become detrimental as an organism ages), and accumulation of damaged proteins (Gladyshev, 2016). Whatever the actual cause of biological aging, aging is associated with several detrimental processes including DNA damage, impaired autophagy (a cell biological process important for clearance of damaged organelles (Martinez‐Lopez *et al*., 2015), and elevated oxidative stress due to mitochondrial dysfunction (Fridovich, 2004; Wang & Bennett, 2012; Tchkonia *et al*. 2013; Palmer *et al*., 2015). Interestingly, HF is associated with similar cellular perturbations, which suggests that HF could be considered as an accelerated form of aging (Dutta *et al*., 2012; Wohlgemuth *et al*., 2014). Thus, it is possible that the processes that underlie both frailty and HF could each perturb homeostasis to lead to low‐level chronic inflammation.

**Figure 1:**
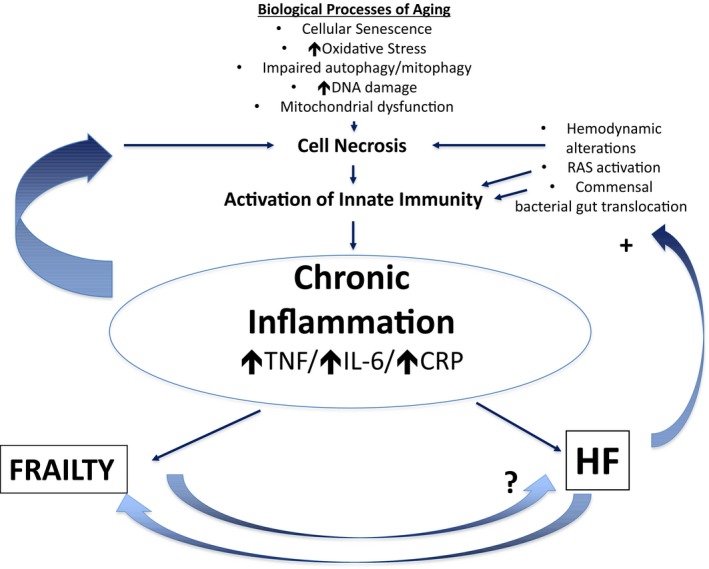
Possible inflammatory pathophysiological link between frailty and HF processes that occur with aging (e.g., cellular senescence, increased oxidative stress, reduced autophagy or mitophagy, increased DNA damage, or mitochondrial dysfunction) accompany both frailty and HF. These processes may disrupt cellular homeostasis and lead to cell death. Cell death activates the innate immune system to induce inflammation manifest as circulating inflammatory cytokines. Subsequent inflammation may then exacerbate the cellular processes mentioned above to perpetuate cell death, in a positive feedback loop. HF may also exacerbate chronic inflammation independently of the above processes, by hemodynamic compromise, activation of the renin–angiotensin system, and translocation of gut bacterial commensals into the systemic circulation. HF likely induces frailty symptoms, but it is less clear whether frailty predisposes to HF.

The etiology of inflammation that leads to frailty may be shared or independent to HF. It is likely that HF leads to frailty, but whether frailty that precedes HF causes cardiac dysfunction is not clear (Fig. 1). DNA damage, impaired autophagy, and mitochondrial dysfunction are biological processes that occur in both aging and HF, and these processes can lead to metabolic dysfunction, cellular senescence, and ultimately cellular necrosis, leading to activation of innate immunity and the production of inflammatory mediators into the circulation. The protein STAT3 limits redox stress and promotes mitochondrial function, and mice lacking STAT3 have increased proinflammatory cytokines and cardiac fibrosis with age (Jacoby *et al*., 2003). Additionally, senescence‐prone mice have elevated levels of pro‐inflammatory cytokines including IL‐1β, a signature cytokine of inflammasome activation (Saito & Papaconstantinou, 2001; Karuppagounder *et al*., 2016). Aged mice (24 months of age) deficient in the NLRP3 inflammasome exhibit enhanced walk distance and running time as compared to their wild‐type controls, suggesting that NLRP3 may enhance inflammation that leads to frailty (Youm *et al*., 2013). Interestingly, there is emerging evidence of NLRP3 activation in HF patients (Butts *et al*., 2015). Hence, the NLRP3 inflammasome may be a common pathway by which frailty and HF interact, although definitive proof will require future investigation.

Dysregulated mitophagy also fails to maintain a pool of high‐quality mitochondria, and the mitochondrial oxidative phosphorylation and membrane potential per mitochondrion increase to meet cellular demand (Twig & Shirihai, 2011). This leads to greater production of ROS and damage to mitochondrial proteins, lipids, and DNA which further activates innate immunity and chronic inflammation (Baroja‐Mazo *et al*., 2014; Wu *et al*., 2015). These processes contribute to cardiomyocyte dysfunction, which occurs with aging and could, in turn, lead to chronic inflammation, remodeling within the myocardium, and pathological left ventricular hypertrophy to exacerbate HF regardless of etiology (Tsutsui *et al*., 2009; Hafner *et al*., 2010; Hoshino *et al*., 2013). As aging is linked to both frailty and HF, it is conceptually plausible that the impaired biological processes that lead to chronic inflammation serve as a common pathophysiological link between frailty and HF (Fig. 1).

As cells die, they release intracellular contents, such as DNA that activate inflammation. An experimental study found that defects in clearing defective mitochondria (i.e., a process termed mitophagy Lemasters, 2005) within the myocardium leads to mitochondrial dysfunction (Hoshino *et al*., 2013). Furthermore, defective mitophagy within the myocardium leads to cell death and the release of mitochondrial DNA that activates Toll‐like receptor (TLR) 9 to induce myocardial inflammation, pressure overload, and cardiac hypertrophy leading to HF (Oka *et al*., 2012). Defective mitophagy also leads to accumulation of dysfunctional mitochondria which produce more proinflammatory signals, termed mitochondrial damage‐associated molecular patterns (Zhang *et al*., 2010; Dall'Olio *et al*., 2013). Skeletal muscle from older adults has reduced ATP production, maximal bioenergetic capacity, and mitochondrial content compared to younger counterparts (Short *et al*., 2005), and cardiac muscle oxidative phosphorylation capacity is reduced in the earliest stages of heart failure in humans (Diamant *et al*., 2003; Hepple, 2016). Skeletal muscle oxidative capacity, mitochondrial content, and fusion are abnormal in older patients with HFpEF (Molina *et al*., 2016). Thus, a potential unifying model for a common pathophysiological pathway between frailty and HF may be impaired mitophagy and mitochondrial dysfunction within cardiomyocytes and skeletal muscle, causing cell death and activation of innate immunity to induce chronic, low‐grade systemic inflammation.

As HF may enhance frailty, another model for the interaction between HF and frailty is that HF precedes or exacerbates frailty. The hemodynamic alterations that occur with HF could induce tissue hypoxia, with resulting cell death and inflammation (Fig. 1). Furthermore, intestinal ischemia from poor gut perfusion and/or congestion related to volume overload, as discussed above, could lead to translocation of gut bacterial commensals, which could activate innate immunity to heighten systemic inflammation and increase risk for HF (Rogler & Rosano, 2014). Finally, neurohormonal pathways, such as the renin–angiotensin system via activation of the sympathetic nervous system, which is induced by HF, can also activate innate immunity (Mann, 2015) to induce low‐grade sterile inflammation (Kalra *et al*., 2002).

As frailty can occur without end‐stage disease, HF is not required for frailty as stated above. Whether frailty mechanistically leads to end‐organ disease such as HF is not known (Fig. 1). It is also plausible that the chronic inflammation that occurs with frailty has a distinct origin from that of HF. One can speculate that cellular senescence in extra‐cardiac sites, such as adipose tissue, lymphoid system, or the vasculature, may lead to chronic inflammation. The subsequent inflammation could lead to frailty and could also negatively effect myocardial function ‘from a distance’ via the negative inotropic effects of the circulating cytokines (Mann, 2015). Interestingly, chronic inflammation and associated vascular dysfunction have also recently been linked to HFpEF (Paulus & Tschope, 2013; Glezeva *et al*., 2015; Franssen *et al*., 2016), the most common form of HF in the older adults (Upadhya *et al*., 2015). Systemic inflammation can also accelerate skeletal muscle apoptosis and promote sarcopenia (Muscaritoli *et al*., 2010). Conceivably, this could enhance immobility and cachexia associated with both HF and frailty. Clearly, future research will be required to decipher the role inflammation plays in the complex interaction between HF, frailty, and cachexia (Hubbard *et al*., 2008).

## How to examine the mechanisms of frailty experimentally

One of the challenges in delineating the underlying pathophysiology of frailty is that, unlike HF (Patten & Hall‐Porter, 2009), there are limited experimental models to investigate the pathophysiology of frailty, at least frailty that is not accompanied by end‐organ disease. Development of such models would allow one to elucidate the etiology of elevated inflammatory proteins that occur with frailty and determine whether the underlying pathophysiology of frailty is unique or shared with the pathophysiology of end‐organ disease such as HF. It may also allow one to determine whether an inflammatory mediator is causal to frailty or merely a result of frailty. Cause–effect relationship will be important, given the result of clinical studies that found that inhibiting certain inflammatory cytokines, for example, TNF‐α, had no impact on mortality in the setting of HFrEF (Coletta *et al*., 2002). Investigators have found that mice that are deficient in IL‐10, an immune suppressive cytokine, exhibit some clinical features of frailty, specifically declining muscle strength by 14 months of age as compared to age‐matched wild‐type controls (Walston *et al*., 2008). This study represents a potential murine model of frailty; however, it is confounded as IL‐10‐deficient mice exhibit signs of inflammatory bowel disease (Davidson *et al*., 2000), which could explain the features described in the study. Recently, a frailty index in aging wild‐type C57BL/6 strain mice has been developed by measuring 31 health‐related variables including mobility, hemodynamic, metabolic, and body composition parameters both invasively (Parks *et al*., 2012) and more recently with noninvasive approaches (Whitehead *et al*., 2014). Furthermore, others have robustly characterized physical function measures in mice to standardize measures which could provide index values for physical function to aide in establishing murine models of frailty (Justice *et al*., 2014). If measurements of chronic inflammation can be accurately assessed in wild‐type aging mice and correlated with this frailty assessment, then it may be possible to develop an experimental model to mechanistically investigate the inflammatory pathophysiological basis of frailty.

## Leveraging therapies for HF—a potential opportunity to inform of the biology of frailty and HF?

There is accumulating evidence that endurance and resistance exercise training improve functional capacity and reduces hospitalizations in HF patients (Gillespie *et al*., 2012; Kitzman *et al*., 2016). The Heart Failure: A Controlled Trial Investigating Outcomes of Exercise Training (HF*‐*ACTION) trial demonstrated favorable outcomes in patients with HFrEF although the effects of exercise training on formal measures of frailty and markers of inflammation were not studied in HF‐ACTION (O'Connor *et al*., 2009). One potential mechanism of benefit with exercise training is reduction of inflammation within skeletal muscle (Addison *et al*., 2012), and importantly, a prior study found that exercise training reduced local inflammation within the skeletal muscle (manifest by iNOS protein levels) in patients with HFrEF (Gielen *et al*., 2005). Another recent study found that in patients with HFpEF, there was a reduction in mitochondrial content within the skeletal muscle (Molina *et al*., 2016; b), although this study did not correlate this to inflammation. It was shown that exercise‐induced increase in cardiorespiratory fitness was associated with reductions in CRP in patients with HFpEF (Kitzman *et al*., 2016). It will thus be important for future investigation to determine whether improvements in frailty with exercise training correlate with reduction in either local inflammation within the skeletal muscle or systemic inflammation within the circulation.

Currently, there are ongoing clinical studies to assess whether advanced therapies for HF (e.g., left ventricular assist device [LVAD] implantation) improve frailty symptoms (clinicaltrials.gov NCT02156583) or whether improving frailty symptoms prior to resolving the hemodynamics of HF improve outcomes after transcatheter aortic valve replacement (TAVR; clinicaltrials.gov, NCT02597985). Few studies have examined whether LVAD impact inflammatory cytokine levels, with one revealing that LVADs may reduce circulating IL‐6 and TNF‐α levels 1 month after implantation (Clark *et al*., 2001), but another showing that CRP and IL‐8 (a neutrophil attracting chemokine) increase postimplantation (Grosman‐Rimon *et al*., 2014). Interestingly, even medical management of acute decompensated HF can reduce innate immune activation (Goonewardena *et al*., 2015). The relationship between alterations in cytokines post‐LVAD implantation or acute treatment of decompensated HF and frailty has not yet been investigated and should be the focus of future investigation.

As increasing numbers of older frail patients receive advanced options for end‐stage HF, there is a unique opportunity to perform translational research that could inform on the inflammatory biological basis of frailty and how it interacts with HF. LVADs improve cardiac hemodynamics to resolve the poor tissue perfusion that occurs with HF. Thus, investigation of frail patients with HF that receive LVADs could inform on the contribution of end‐organ dysfunction to the frailty clinical and inflammatory phenotypes. Future clinical investigation is required to determine whether resolving the hemodynamic alterations of HF using advanced therapeutic options would improve the systemic inflammatory profile associated with both frailty and HF.

Given the anticipated rise in the number of older people with HF and current limited experimental models of frailty, there is a rare opportunity to leverage ongoing clinical practice to yield novel insights as to whether therapies that improve HF resolve the chronic inflammation that occurs in frailty and HF. Such lines of investigation could provide precious information on the biological underpinnings of frailty and end‐organ disease such as HF.

## Funding

Daniel Goldstein has following NIH grant funding: AG050096, HL 130669, and AG028082. Scott Hummel has following NIH grant funding: HL‐K23109176.

## Conflict of interest

None declared.
